# Recent Discoveries on the Involvement of Krüppel-Like Factor 4 in the Most Common Cancer Types

**DOI:** 10.3390/ijms21228843

**Published:** 2020-11-22

**Authors:** Agnieszka Taracha-Wisniewska, Grzegorz Kotarba, Sebastian Dworkin, Tomasz Wilanowski

**Affiliations:** 1 Institute of Genetics and Biotechnology, Faculty of Biology, University of Warsaw, 1 Miecznikowa St., 02-096 Warsaw, Poland; ataracha@biol.uw.edu.pl (A.T.-W.); g.kotarba@biol.uw.edu.pl (G.K.); 2 Department of Physiology, Anatomy and Microbiology, School of Life Sciences, La Trobe University, Bundoora, VIC 3086, Australia; s.dworkin@latrobe.edu.au

**Keywords:** breast cancer, colorectal cancer, gastric cancer, hepatocellular carcinoma, KLF4, lung cancer, microenvironment, prostate cancer, subcellular localization, transcription factor

## Abstract

Krüppel-like factor 4 (KLF4) is a transcription factor highly conserved in evolution. It is particularly well known for its role in inducing pluripotent stem cells. In addition, KLF4 plays many roles in cancer. The results of most studies suggest that KLF4 is a tumor suppressor. However, the functioning of KLF4 is regulated at many levels. These include regulation of transcription, alternative splicing, miRNA, post-translational modifications, subcellular localization, protein stability and interactions with other molecules. Simple experiments aimed at assaying transcript levels or protein levels fail to address this complexity and thus may deliver misleading results. Tumor subtypes are also important; for example, in prostate cancer KLF4 is highly expressed in indolent tumors where it impedes tumor progression, while it is absent from aggressive prostate tumors. KLF4 is important in regulating response to many known drugs, and it also plays a role in tumor microenvironment. More and more information is available about upstream regulators, downstream targets and signaling pathways associated with the involvement of KLF4 in cancer. Furthermore, KLF4 performs critical function in the overall regulation of tissue homeostasis, cellular integrity, and progression towards malignancy. Here we summarize and analyze the latest findings concerning this fascinating transcription factor.

## 1. Introduction

Krüppel-like factor 4 (KLF4) is a transcription factor very important in various developmental processes and disease states. Although the most recent review of its key roles in development, cellular reprogramming and cancer appeared in 2017 [1], since then many new reports have explored the roles played by KLF4 in cancer. These discoveries shed new light on the functioning, regulation and significance of KLF4 in various types of cancer. The emerging picture is very complex and indicates that many aspects of KLF4 functioning must be taken into account before any conclusions can be made. On the other hand, many results are very promising, not only from the point of view of basic research, but also because they can potentially find clinical applications. In the present review we focus on the role of KLF4 in the most common types of cancer [2], as the vast majority of recent reports are concerned with these cancer types.

Traditionally, the role of KLF4 in cancer has been to act primarily as a tumor suppressor, that is, to drive terminal differentiation and inhibit cellular proliferation. However, recent studies analyzing data from cancer patients, in vitro tissue and cell culture experiments, murine models of metastasis and also of conditional (tissue-specific deletion) animal models indicate that the role of KLF4 is actually much more extensive than originally believed, and is extremely dependent on the microenvironment in which KLF4 drives its cadre of transcriptional targets. Moreover, recent findings (primarily from *Cre-lox* dependent *Klf4*-deletion) indicate that loss of KLF4 acts as a “sensitizing” mutation, in that tissue homeostasis is often only marginally perturbed, however when a further stressor (such as environmental factor/toxin exposure, further genetic mutation etc.) is applied, the disruptions to tissue homeostasis are far more pronounced than they are in tissues with normal KLF4 function. In this way, KLF4 may be considered to act not only as a tumor suppressor, but more broadly, as a critical “cell stability molecule”, and an important maintainer of tissue homeostasis.

## 2. Colorectal Cancer

The broadly described role of KLF4 in colorectal cancer (CRC) remains controversial. Many studies have shown that KLF4 plays a tumor-suppressive role in CRC [3]. The reduced expression of KLF4 in human CRC tissues has been associated with increased growth of CRC cells, lymphatic node metastasis, reduced tumor cell differentiation, and tumor recurrence. CRC patients with lymph node metastasis display reduced KLF4 expression. Furthermore, the downregulation of KLF4 is associated with poor prognosis in human CRC patients, decreased overall survival as well as disease-free survival [3].

KLF4 inhibits CRC cell proliferation through upregulation of N-Myc downstream regulated gene 2 (NDRG2) by binding to the *NDRG2* promoter. Lower expression of KLF4, as well as NDRG2, in CRC patients was correlated with poor overall survival [4]. KLF4 acts as transcriptional repressor of GINS complex subunit 4 (GINS4), a prognostic biomarker promoting the growth of CRC. The expression of GINS4 is significantly elevated in CRC tumor samples [5].

Loss of KLF4 in CRC tissues is associated with epithelial-mesenchymal transition (EMT). There is a marked decrease in KLF4 expression in CRC tumor samples obtained from patients, which is also observed in the mouse model. The same study has shown a negative correlation between KLF4 levels and mesenchymal markers both in human patients and in mice treated with azoxymethane and dextran sodium sulfate (AOM/DSS). These markers include TWIST, β-catenin, claudin-1, N-cadherin, SNAI2 and vimentin. in CRC patient tumor sections. However, the expression of KLF4 is positively correlated with the epithelial marker E-cadherin [6].

The intestinal epithelium-specific deletion of *Klf4* in mice increases genetic instability and accelerated progression of colitis-associated colorectal cancer (CAC). Mice with intestinal epithelium-specific deletion of *Klf4* (*Klf4^ΔIS^*) treated with AOM and DSS developed significantly more adenomatous polyps and carcinomas in situ in comparison to treated control *Klf4^fl/fl^* mice. The tumors and polyps in these mice display an increased number of mitotic cells with more than 2 centrosomes [7]. On the other hand, the expression of KLF4 is specifically increased in colorectal epithelial cancer cell lines, Caco-2 and HCT116, but not in the other human colorectal epithelial cell lines. Overexpression of KLF4 was induced in the HCT166 cell line with the help of small activating RNAs. This promoted migration and invasion of cells. It was found that the underlying molecular mechanism included the induction of EMT and nuclear translocation of β-catenin [8].

The analysis of cell proliferation and tissue remodeling from the cohort of colorectal cancer patients have also predicted KLF4 to be a driver of tissue remodeling in CRC via myeloid cell infiltration [9]. KLF4 can also indirectly modulate the actin cytoskeleton morphology via activity of RhoA in order to inhibit cellular migration and invasion of the human colon cancer cell line RKO [10].

The well-described role of microRNA in colorectal cancer and its significance in cancer prognosis and treatment was reviewed elsewhere [11]. The relationship between some specific microRNAs and KLF4 in these neoplasms is also well known. KLF4 is a direct target of miR-543, miRNA highly expressed in CRC samples and cell lines, and associated with tumor size, TNM stage and metastasis. These studies have shown an obvious inverse correlation between miR-543 and KLF4 expression in CRC tissues. By targeting KLF4, miR-543 facilitates colorectal cancer proliferation and metastasis [12]. MiR-25-3p, miR-103 and miR-107, all promote metastasis of CRC by targeting KLF4 [13]. Furthermore, miR-25-3p also regulates KLF4 in endothelial cells, as it can be transferred into them from CRC cells via exosomes. By targeting KLF2 and KLF4, miR-25-3p regulates the expression of VEGFR2, ZO-1, occludin and claudin-5, and in this way it promotes vascular permeability and angiogenesis [14].

MiR-7-5p negatively regulates KLF4 which results in increased proliferation and migration of CRC cells Moreover, KLF4 overexpression rescued the suppressive effects of miR-7-5p on CRC cell proliferation and migration [15]. MicroRNA-10b, a key regulator of metastasis in many human tumors, regulates KLF4 expression and in this way it controls the metastasis and proliferation of CRC cells [16]. KLF4 is directly involved in the regulation of miR-153-1 expression. The long non-coding RNA, Taurine up-regulated 1 (TUG1), negatively regulates KLF4 expression. TUG1 interacts with EZH2 (enhancer of zeste 2 polycomb repressive complex 2 subunit). This regulation contributes to the growth, metastasis and EMT of CRC in mice in vivo [17].

KLF4 regulates stemness and mesenchymal properties of CRC stem cells through the TGF-β1/Smad/snail pathway in Lgr5^+^CD44^+^EpCAM^+^ colorectal cancer stem cells (CSCs), which are responsible for initiating and sustaining tumor development and progression [18]. It appears that KLF4 participates in the response of cancer cells to chemotherapy. KLF4 is very important for maintaining the stemness in cancer cells. KLF4 enhances the expression of survival proteins hTERT and HMGB1 (high mobility group box 1) which sensitizes cancer cells to cisplatin cytotoxicity. In the presence of cisplatin, expression of HMGB1 and hTERT is negatively regulated by KLF4. What is more, KLF4 promotes the cisplatin-mediated G2/M cell cycle arrest while a knock-down of KLF4 induces cisplatin-mediated S-phase arrest compared to control. In cisplatin-treated and KLF4 knock-down HCT-15 cells, compared to the empty vector control, the level of reactive oxygen species was decreased, accounting for increased cell survival. Therefore it appears that increasing KLF4 expression might sensitize drug-resistant cancer cells to chemotherapy [19]. Sijunzi decoction is a traditional Chinese medicine product used in the prevention and treatment of CRC. KLF4 is a likely molecular target of this medical product [20].

KLF4 mediates the effects of mesalazine, also known as 5-aminosalicylic acid (5-ASA), an aminosalicylate anti-inflammatory drug [21], on the β-catenin pathway in colon cancer cells. The treatment with 5-ASA–induces µ-protocadherin expression, and KLF4 is a direct regulator of µ-protocadherin in this context. The underlying molecular mechanism involves miR-130a and miR-135b, as these microRNAs target KLF4 and 5-ASA treatment suppresses their expression [22]. KLF4 p.A472D mutation contributes to acquired resistance to cetuximab, a human-mouse chimeric IgG1 mAb that targets the extracellular domain of epidermal growth factor receptor (EGFR) and is effective in treating RAS wild-type and BRAF V600E wild-type patients with metastatic CRC [23].

In HCT116RR, derived radio-resistant cancer cells, KLF4 directly interacts with the human telomeric RAP1 protein [24]. The silencing of RAP1 reverses the radio-resistant phenotype in these cells and increases their sensitivity to radiotherapy. Increased RAP1 levels were associated with a poor survival rate, indicating that RAP1 could serve a marker for survival prediction in these types of cancer [24], although the precise relationship between RAP1 and KLF4 needs to be investigated further. B-cell-specific Moloney murine leukemia virus insertion site 1 (BMI1) deficiency sensitizes cells to radiation treatment by modulating the expression of KLF4 and leads to enhanced radiosensitivity in microsatellite stable colorectal cancers [25]. In summary, KLF4 serves as a tumor suppressor in CRC and sensitizes CRC cells to various forms of treatment. It seems to be involved in a wide variety of molecular pathways and cellular processes. The overall picture of these interactions is very complicated and calls for further research to unravel all its nuances.

## 3. Breast Cancer

The role of KLF4 in breast cancer is complex; it has been reported that KLF4 has dual function as either a tumor suppressor or an oncogene, in a context-specific manner. Recent work revealed that in triple-negative breast cancer (TNBC) KLF4 is a repressor of the *EGFR* gene leading to a decrease in both total and phosphorylated EGFR levels in MDA-MB-231 and MDA-MB-468 cells. Furthermore, overexpression of KLF4 inhibits migration, invasion and growth of TNBC cells in vitro and increases the sensitivity of these cells to erlotinib [26]. Additionally, the group of TNBC patients with high KLF4 expression have more favorable prognostic factors (overall survival and disease-free survival rates) than patients characterized with low KLF4 expression [27]. It should be noted that KLF4 is a favorable prognostic indicator for patients with other subtypes of breast cancer as well (classified on the basis of the estrogen receptor (ER) and HER2 status) [28]. Lu et al. investigated a novel mechanism of KLF4 regulation in breast cancer cells, involving covalent head-to-tail looped RNA, originating from the euchromatic histone lysine methyltransferase 1 (circEHMT1). They found that KLF4-dependent inhibition of migration and invasion of breast cancer cells is regulated by miR-1233-3p which is a target of circEHMT1 [29]. Other studies revealed interesting mechanisms of KLF4 regulation in breast cancer cells, involving DEAD-BOX (DDX) RNA helicase (DDX3X). Data showed that DDX3X directly interacts with *KLF4* mRNA and negatively regulates its splicing. The DDX3X knockdown in MCF7 cells drives the cell cycle arrest by increasing KLF4 protein levels [30].

Nuclear factor I-C (NFI-C) appears to be an essential factor for the maintenance of epithelial differentiation and inhibits EMT and metastasis of breast cancer cells by regulating KLF4. NFI-C directly interacts with the *KLF4* promoter and stimulates its transcriptional activity which in consequence induces mesenchymal-epithelial transition (MET) [31]. Importantly, KLF4 is a key inducer of MET in normal mammary epithelial cells and breast cancer cells, through its ability to activate the epithelial program by triggering E-cadherin expression [32]. Other mechanisms suggesting protective role of KLF4 in breast cancer involve human 1-acylglycerol-3-phosphate O-acyltransferase 9 (AGPAT9). AGPAT9 inhibits breast cancer cell proliferation, migration and invasion both in vitro and in vivo through the KLF4/*Homo sapiens* longevity assurance homolog 2 of yeast LAG1 (LASS2)/ vacuolar-H^+^-ATPase (V-ATPase) signaling pathway [33]. Results showed that the LASS2 expression is activated by KLF4 and LASS2 is its target gene. Moreover, the LASS2 inhibition of the V-ATPase activity occurs through LASS2 interaction with the c subunit of the V-ATPase proton pump (ATP6V0C) [34,35]. The above findings indicate that KLF4 suppresses breast cancer development. Conversely, there are also reports suggesting that KLF4 plays an oncogenic role in mammary tumorigenesis. An in vitro and in vivo study performed by Zhou and colleagues showed that breast cancer cell metastasis is promoted by ATXN3 (Ataxin-3, ATX3, AT3 or MJD), which is a novel deubiquitinating enzyme of KLF4 [36]. They also found that a member of the F-box protein family (FBXO32) mediates KLF4 ubiquitination and degradation, and in consequence suppresses breast cancer tumorigenesis [37].

In the 12-O-tetradecanoylphorbol-13-acetate (TPA)-induced carcinogenesis model KLF4 expression is up-regulated. Furthermore, KLF4 can bind to the promoter of S100 calcium binding protein A14 (*S100A14*) gene, increasing its mRNA and protein levels, which promotes breast cancer cell motility [38]. The study of the role of KLF4 in glycolytic metabolism and proliferation in breast cancer cells revealed that KLF4 is a stimulator of glycolytic metabolism. KLF4 directly binds to the phosphofructokinase platelet gene (*PFKP*) promoter and activates its transcription, while KLF4 knockdown decreases PFKP expression resulting in reduced glucose uptake and lactate production in vitro. Additionally, there is a statistically significant positive correlation between KLF4 and PFKP expression in breast cancer tissues [39].

The expression of KLF4 is significantly and inversely correlated with brain, but not bone, metastasis-free survival [40]. Using a mouse model it was demonstrated that miR-7-2 suppresses brain metastasis by inhibiting KLF4 expression. In addition, further in vitro experiments showed that miR-7 reduces the ability of invasion and self-renewal of cancer stem cells (CSCs) by modulating KLF4 expression [40]. In agreement with these findings the silencing of WNT1-inducible signaling pathway protein 2 (WISP2) signaling in human breast adenocarcinoma MCF7 cells resulted in miR-7 inhibition and elevation of KLF4 expression. The above mechanism is responsible for the reduction in breast cancer cells susceptibility to the cytotoxic T-lymphocyte (CTL)-mediated lysis [41].

Other studies revealed that dual specificity tyrosine phosphorylation regulated kinase 2 (DYRK2) negatively regulates the formation of breast CSCs, and KLF4 is a key mediator in this process. Moreover, androgen receptor (AR) activates KLF4 expression by binding to the *KLF4* promoter and this process is DYRK2-dependent [42]. KLF4 may influence tumor response to chemotherapy. KLF4 regulates chemoresistance in breast cancer cells. Cisplatin treatment elevates KLF4 protein levels, which led to reduced sensitivity of breast cancer cells to this drug [43]. In addition, patients with locally advanced breast cancer with high KLF4 expression have lower pathologic complete remission (pCR) rates after neoadjuvant chemotherapy [44]. Thus the overall picture of KLF4 involvement in breast cancer is even more complicated than in CRC. As in CRC, KLF4 serves as a tumor suppressor in breast cancer and sensitizes breast cancer cells to various forms of treatment, but it can also act as a tumor promoting factor in breast cancer. There are many molecular pathways and cellular processes responsible for the involvement of KLF4 in breast cancer. Certainly, more studies are necessary to shed more light on this topic.

## 4. Hepatocellular Carcinoma

According to the latest findings, in hepatocellular carcinoma (HCC) KLF4 performs a tumor suppressive role [45,46,47]. It inhibits proliferation, migration, invasion and EMT of HCC cells [45]. The expression of KLF4 is reduced in HCC tumors, in comparison with the surrounding non-tumorous tissues, and is negatively correlated with the number of tumors, grades of differentiation, and stages of LNM (lymph node metastasis) and TNM (tumor node metastasis) [45,48]. High KLF4 levels in tumor tissues are associated with both better overall survival rate and recurrence-free survival rate, while low KLF4 expression may mean a poor prognosis for HCC patients [45,48]. KLF4 may thus become not only a valuable prognostic biomarker but may also be a therapeutic target in HCC [45,48,49].

KLF4 is very unstable in living cells. Its half-life is only about two hours, as it is rapidly ubiquitinated and degraded in proteasomes [50]. In HCC, this process is regulated by tumor necrosis factor receptor-associated factor 7 (TRAF7), which acts as an E3-ubiquitin ligase. TRAF7 promotes HCC migration and invasion through ubiquitination and subsequent degradation of KLF4 [51]. KLF4 expression in HCC is negatively regulated by a number of microRNAs: miR-9-5p, miR-10b, miR-18a and miR-124 [52,53,54,55]. Histone methyltransferase SET8 binds to KLF4 and suppresses its expression [46]. Subsequently, KLF4 redirects carbohydrate flux from glycolysis to mitochondrial respiration. The underlying molecular mechanism involves the activation of sirtuin 4 (SIRT4) expression by KLF4, which binds directly to the *SIRT4* promoter and positively regulates its expression [46]. KLF4 activity as a transcriptional transactivator is negatively regulated by DEAD box RNA helicase 17 (DDX17), which displays a tumor promoting function in HCC [56]. 

The expression of KLF4 can also be regulated at the level of splicing. Splicing factor 3b subunit 4 (SF3B4) is frequently overexpressed in HCC samples, where it promotes cancer development [57]. At the molecular level, SF3B4 overexpression triggers SF3B complex to splice *KLF4* primary transcript to nonfunctional skipped exon mature transcripts [57]. All the above findings indicate that the mechanisms of regulation of KLF4 activity are complex, and that simply measuring the levels of KLF4 expression is insufficient to appropriately investigate its involvement in HCC.

Monoglyceride lipase (MGLL; EC 3.1.1.23) is one of the targets of KLF4 regulation relevant for the development of HCC [58]. The expression of MGLL is decreased in HCC samples, both at the mRNA and protein levels [59]. Patients with low MGLL expression have lower 5-year overall survival rate, and overexpression of MGLL suppresses HCC cell migration [58]. KLF4 directly binds to the *MGLL* promoter and positively regulates the expression of *MGLL* in HCC cells [59]. KLF4 also directly binds to the promoter of the gene coding for Ring1- and YY1-binding protein (RYBP), a tumor suppressor, and positively regulates its expression [60]. miR-31 is yet another direct target of KLF4 regulation in HCC [61]. KLF4 positively regulates the expression of tetraspanins CD9 and CD81 [62]. These proteins are surface markers of exosomes, and they act as tumor suppressors in HCC where they inhibit cell proliferation by negatively regulating the MAPK/JNK signaling pathway [62]. 

KLF4 represses the expression of another Krüppel-like factor, KLF11, by directly binding to its promoter, whereas KLF11 inhibits the expression of Smad7 through direct binding to its promoter, and this in turn triggers EMT in HCC cells [55]. Interestingly, KLF4 can also directly bind to the Smad7 promoter but, unlike KLF11, it positively regulates its transcription [47]. In this way KLF4 suppresses oncogenic transforming growth factor beta (TGF-β) signaling, and therefore loss of KLF4 expression in primary HCC cells may contribute towards the activation of oncogenic TGF-β signaling and subsequent tumor progression [47]. KLF4 positively regulates the expression of P-cadherin, which acts as a tumor suppressor in HCC [63]. P-cadherin functions in HCC by modulating glycogen synthase kinase 3 beta (GSK-3β) signaling, thus adding yet another signaling pathway to those influenced by KLF4 [63].

Increased expression of KLF4 in HCC cells contributes towards their resistance to sorafenib, a protein kinase inhibitor approved for the treatment of HCC [64]. KLF4 and epidermal growth factor receptor (EGFR) constitute a positive feedback loop, where KLF4 directly binds to the *EGFR* promoter and positively regulates its transcription, while nuclear EGFR directly binds to the *KLF4* promoter and increases its transcription. However, the underlying molecular mechanisms remain elusive. KLF4 might induce the resistance to sorafenib by inducing CSCc, because CSCs have strong chemoresistance to antitumor agents [64]. KLF4 is a well-known Yamanaka factor, one of four core factors known to possess the ability to “re-program” differentiated cells into a more immature state, and its ectopic expression can reprogram various differentiated cells to pluripotent stem cells [65]. The overexpression of KLF4 in the HCC cell line HuH7 can induce a CSC-like phenotype in non-CSC cells by upregulating the expression of EpCAM (epithelial cell adhesion molecule) and CD133/Prominin-1 [66]. However, these latter studies were carried out in only one cell line and, as the authors agree, their investigations will have to be repeated in a series of HCC cell lines with different genetic and epigenetic backgrounds before any far-reaching conclusions can be proposed.

## 5. Lung Cancer

KLF4 is an important suppressor of lung cancer [67,68,69,70]. It inhibits migration, invasion and metastasis of non-small cell lung cancer (NSCLC) cells by attenuating TGF-β1-induced EMT and inhibiting the c-Jun-NH_2_-terminal kinase signaling pathway [68]. However, as the Authors of the latter study acknowledge, the mechanistic explanation for the observed phenomena is lacking [68].

In lung adenocarcinoma, which is one of the subtypes of NSCLC, the activity of KLF4 is regulated at the level of protein stability. Loss of ubiquitin-specific peptidase 10 (USP10) promotes lung tumorigenesis via the downregulation of KLF4 [71]. At the molecular level, USP10 deubiquitinates KLF4 and in this way it blocks KLF4 degradation. KLF4 directly activates the expression of the gene coding for tissue inhibitor of metalloproteinases 3 (TIMP3), which acts as a tumor suppressor [71].

Subcellular localization of KLF4 is very important in NSCLC [72]. KLF4 can be found in both the cytoplasm and the nucleus. The overall survival rate is the highest in patients with high KLF4 levels in the cytoplasm and low KLF4 levels in the nucleus. Cytoplasmic and nuclear levels of KLF4 were found to be independent risk factors for NSCLC. Patients with low KLF4 levels in cell nuclei had better prognosis than those with high levels, but the difference in prognoses of patients with different cytoplasmic levels of KLF4 was not statistically significant. This work very elegantly demonstrates the importance of investigating subcellular localization of KLF4. In this study it was also shown that increased levels of KLF4 in the cell nucleus may participate in resistance to cisplatin [72].

In NSCLC the expression of KLF4 is negatively regulated by various microRNAs: miR-25, miR-103, miR-145 and miR-3120-5p [69,73,74,75]. KLF4 expression is positively regulated by metastasis-associated lung adenocarcinoma transcript 1 (MALAT1), a long non-coding RNA (lncRNA) that has been demonstrated to function as an oncogene [74]. This regulation is indirect as MALAT1 directly targets miR-145 and in this way it reduces the inhibitory effects of miR-145 on KLF4 activity [74]. Thyrotropin Releasing Hormone Degrading Enzyme (TRHDE)-Antisense RNA 1 (TRHDE-AS1) is another lncRNA positively regulating the expression of KLF4, and this regulation is most likely mediated by miR-103 [75]. These findings emphasize the importance of non-coding RNAs in the regulation of KLF4 activity.

KLF4 is negatively regulated by NAD-dependent deacetylase sirtuin 6 (SIRT6), a promoter of metastasis in NSCLC [76]. The molecular mechanism of this regulation involves the Snail transcription factor: SIRT6 deacetylates Snail and prevents its proteasomal degradation, while Snail directly binds to the *KLF4* promoter and represses its transcription [76]. Placenta-specific 8 (PLAC8) and matrix metalloproteinase 2 (MMP2) act as oncogenes in NSCLC, and KLF4 negatively regulates their expression by directly binding to their promoter regions [70,77].

Sulfonylurea receptor 1 (SUR1) is the regulatory subunit of ATP-sensitive potassium channels, and it acts as a tumor promoter in NSCLC [78]. Glibenclamide, a well-established anti-diabetic medication, is a small molecule inhibitor of SUR1. Glibenclamide displays anti-tumor activity in NSCLC cell lines as well as in lung cancer xenografts in nude mice. The molecular mechanism of glibenclamide action involves KLF4, as SUR1 directly interacts with p70S6K and upregulates p70S6K phosphorylation and activity, then p70S6K downregulates KLF4 expression by enhancing DNA-methyltransferase 1 (DNMT1)–mediated methylation of the *KLF4* promoter [78]. This finding is very significant as it demonstrates that the expression of KLF4 can be increased by the oral administration of the small molecule drug glibenclamide, at least in this particular context. KLF4 promotes resistance to gefitinib, an EGFR inhibitor, in NSCLC cells with c-Met overexpression [79]. The underlying molecular mechanism involves the regulation of phosphorylation of c-Met and Akt, as KLF4 negatively regulates the expression of β-catenin and inhibits the binding between c-Met and β-catenin [79].

## 6. Gastric Cancer

Low KLF4 expression is negatively associated with overall survival rate in gastric cancer patients and may thus serve as a prognostic marker in this type of cancer [80]. In gastric cancer, the expression of KLF4 is regulated by miR-32, miR-103, miR-135b-5p and miR-155 [81,82,83,84,85]. LncRNA LINC00673 acts as an oncogene in gastric cancer, and it negatively regulates the expression of KLF4 [86]. LINC00673 directly interacts with two RNA- and DNA-binding proteins, EZH2 (enhancer of zeste 2 polycomb repressive complex 2 subunit) and DNMT1. These two proteins bind to the *KLF4* promoter and suppress its transcription, and interaction with LINC00673 significantly increases the capacity of EZH2 and DNMT1 to bind to this promoter [86]. Small nucleolar RNA host gene 5 (SNHG5) is another lncRNA regulating the expression of KLF4 but, unlike LINC00673, SNHG5 positively regulates KLF4 expression [82]. The underlying molecular mechanism is analogous to that employed by MALAT1 in NSCLC (Section 5: Lung cancer): SNHG5 directly binds miR-32 and in this way it reduces the inhibitory effects of miR-32 on KLF4 activity [82].

KLF4 negatively regulates the expression of inhibitor of apoptosis-stimulating protein of p53 (iASPP), podocalyxin-like 1 (PODXL) and serine/threonine kinase 33 (STK33), which all perform oncogenic functions in gastric cancer [87,88,89]. p53 is a transcription factor and a very well-known tumor suppressor; PODXL is an anti-adhesive transmembrane glycoprotein and STK33 is a member of the calcium/calmodulin-dependent kinase family with critical roles in promoting tumor growth and metastasis [87,88,89]. Thus all of the above signaling pathways may be influenced by KLF4 in gastric cancer. In addition, KLF4 negatively regulates the expression of miR-106a, which in turn targets Smad7, which is involved in the TGF-β signaling pathway [90,91].

KLF4 plays an important role in the development of gastric cancer induced by *Helicobacter pylori* [84,85,92]. The expression of KLF4 is strongly reduced in gastric cancer samples [92]; furthermore, it is lower in *H. pylori*-positive tumors in comparison with *H. pylori*-negative (*H. pylori* uninfected) tumors [85]. This reduction in expression is caused by multiple molecular mechanisms. *H. pylori* infection induces the expression of two microRNAs, miR-135b-5p and miR-155, which both target KLF4 [84,85]. *H. pylori* regulates the expression of these microRNAs by the activation of NF-κB proinflammatory signaling and by the *H. pylori* cytotoxin-associated gene A (CagA) [84,85]. Another mechanism of downregulation of KLF4 expression by *H. pylori* infection involves the methylation of *KLF4* promoter. CagA inhibits the expression of Ten-Eleven Translocation 1 (TET1) [92]. TET1 belongs to a family of α-ketoglutarate-dependent enzymes that catalyze active demethylation of 5-methylcytosine [93]. Thus reduction in TET1 levels suppresses demethylation of *KLF4* promoter and reduces *KLF4* expression [92]. Furthermore, loss of KLF4 induces resistance to cisplatin in *H. pylori*-positive gastric cancer [84]. In summary, KLF4 acts as a tumor suppressor in gastric cancer. Induction of gastric cancer by *H. pylori* infection seems to be mediated, at least in part, by the reduction in KLF4 expression. Furthermore, KLF4 sensitizes gastric cancer cells to cisplatin treatment.

## 7. Prostate Cancer

Findings concerning the role of KLF4 in prostate cancer are contradictory. Some reports indicate that KLF4 promotes prostate cancer growth [94,95] while other reports claim the opposite [96,97,98]. Some research teams discovered that KLF4 is upregulated in prostate tumor samples [95] while other reports claim that the levels of KLF4 are in fact reduced in prostate cancer [96,97,98]. In one study, KLF4 was detected primarily in the cytoplasm of non-tumor prostate tissues, and it was suggested that subcellular localization of KLF4 may be an important factor in prostate cancer [98]. Overexpression of KLF4 inhibited proliferation and migration, induced cell cycle arrest and increased E-cadherin expression in prostate cancer cells [98]. According to the Human Protein Atlas [99] (www.proteinatlas.org), KLF4 is not found in prostate cancer samples, which supports its tumor suppressive, rather than tumor promoting, role in this type of cancer. 

Perhaps the above contradictions can be at least partially resolved by the discovery that the levels of KLF4 vary depending on the subtype of prostate cancer [100]. KLF4 is highly expressed in indolent tumors which do not cause any symptoms and do not require any treatment but is absent from aggressive prostate tumors. The reduced expression of KLF4 is associated with the molecular features of aggressive cancers. Accordingly, induction of KLF4 in established tumors reverses their aggressive phenotype [100]. KLF4 thus blocks the malignant transformation and impedes tumor progression. This anti-tumorigenic effect of KLF4 can also be observed in the prostate cancer cells after bone metastasis [101]. KLF4 may therefore be employed to identify patients with indolent prostate cancer who have good prognosis [100].

In prostate cancer, the expression of KLF4 is regulated by miR-7, miR-32-5p, miR-148-3p and miR-152-3p [94,95,102]. KLF4 and miR-7 form an auto-regulatory feedback loop, in which KLF4 positively regulates the expression of miR-7 while miR-7 negatively regulates the expression of KLF4 [94]. In prostate cancer this feedback loop is dysregulated due to the disrupted miR-7 processing, which leads to the overexpression of KLF4, maintaining stemness of prostate cancer stem cells to promote tumor growth [94]. The expression of KLF4 is negatively regulated by lncRNA LINC00673 [96]. LINC00673 binds to the *KLF4* promoter and recruits DNMT1, DNMT3a and DNMT3b. This results in increased methylation status of the *KLF4* promoter and reduced *KLF4* expression [96]. Lysine (K)-specific methyltransferase 2D (KMT2D) is yet another epigenetic modifier that negatively regulates the expression of KLF4 in prostate cancer [103].

KLF4 positively regulates the expression of miR-1, BCL2-interacting killer (BIK) and insulin-like growth factor 2 (IGF2) in prostate cancer [97,102,104]. The latter occurs in response to cisplatin treatment, thus promoting cisplatin-induced apoptosis in prostate cancer [102]. Apoptosis is triggered by miR-32-5p, which is downregulated upon cisplatin treatment and inhibits the expression of KLF4. Following the administration of cisplatin, KLF4 levels are increased, which leads to increased production of BIK and apoptosis [102]. KLF4 is very important in androgen receptor (AR) signaling [97]. Activated AR binds to the *KLF4* promoter to enhance its expression and reciprocally, KLF4 binds to the *AR* promoter to increase its expression. Ectopic expression of KLF4 in androgen-independent prostate cancer cells induces AR expression and decreases cell proliferation, invasion and metastasis [97].

KLF4 is crucial in the microenvironment of prostate cancer. KLF4 contributes to monocyte development, and prostate cancer growth is slowed in the absence of myeloid KLF4 expression [105]. The underlying molecular mechanism involves tumor-associated macrophages and activation of pathways associated with pro-inflammatory states [105].

**Figure 1:**
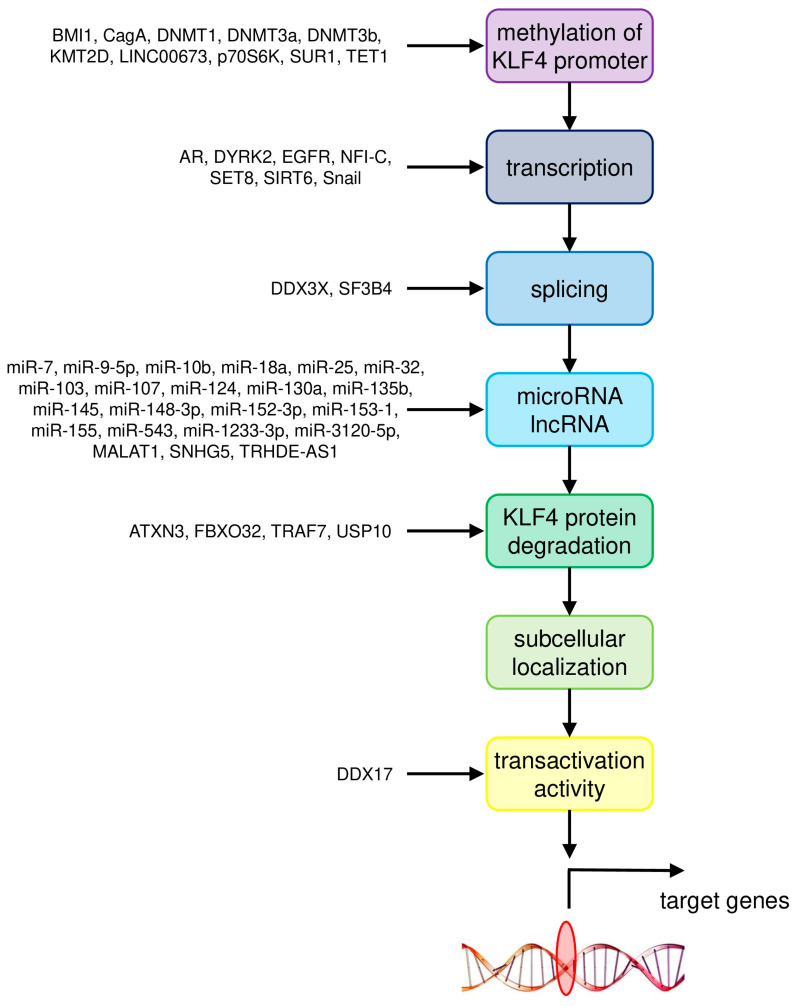
Summary of molecular mechanisms of regulation of KLF4 activity in the most common cancer types.

Therefore the involvement of KLF4 in cancer development is not limited to cancer cells, but it is very important in the tumor microenvironment as well. The molecular mechanisms responsible for the involvement of KLF4 in the most common cancer types are summarized in Table 1. The mechanisms of regulation of KLF4 activity in these cancers are summarized in Figure 1.

## 8. Links between KLF4, Development, Tissue-Specific Conditional Deletion and Cancer

To understand the role that KLF4 plays in cancer, it is important to also understand the role it plays in normal embryogenesis, tissue formation, growth and homeostasis. This is because many processes relevant for cancer development and progression share multiple parallels with the basic principles of normal tissue development and growth. These processes include proliferation, differentiation, cell-cycle control, apoptosis, maintenance of cell “stemness”, maintenance of epithelial vs mesenchymal phenotype and angiogenesis, among others. Therefore by examining animal models in which KLF4 function has been abrogated, we can better understand how these otherwise normal processes may be co-opted, or dysregulated, during the initiation and progression of malignancy.

Mouse models of disrupted KLF4 function have provided substantial insights into its cell-intrinsic roles. As one of the four Yamanaka factors, KLF4 is one of the best-characterized “pluripotency genes” [65]. Complete deletion of *Klf4* leads to rapid embryonic lethality shortly after birth due to extreme dehydration, as a consequence of impaired epidermal differentiation [106]. This is critical, as the imbalance between proliferation and differentiation is an important feature of cancer development and progression. However, the ability of these *Klf4*^−/−^ embryos to survive gestation is likely mitigated somewhat by compensatory upregulation of other, related family members such as KLF2 or KLF5. This theory is predicated on the finding that a transgenic (knock-in) mouse model comprising a glutamylation-defective KLF4 protein (with presumably no compensatory upregulation by related family members), presents with a far more severe phenotype, that is, impaired blastocyst formation by embryonic day (E) 3.5, with embryonic lethality seen well before birth [107]. Irrespective, both these models indicate that complete KLF4 loss in utero is incompatible with life, and concomitant with its role in cancer progression, strongly indicate that this protein is a key element of cellular integrity.

The role of KLF4 in maintaining the homeostasis of cellular compartments that require regeneration and replenishment in adulthood has been thoroughly interrogated through numerous conditional deletion approaches (using the well-validated Cre-lox system to effect tissue specific loss-of-function). Given the critical role of KLF4 in maintaining cellular integrity, one would expect that de-regulation of KLF4 function, therefore, would lead to substantial disruption in cellular architecture, integrity and maintenance, and may even directly lead to cancer. Intriguingly, however, these studies typically reveal only very minor aberrations in tissue maintenance and homeostasis. These somewhat surprising findings indicate that in the post-natal animal, KLF4 is largely dispensable for tissue homeostasis, with animal models comprising conditional deletion typically presenting with minor or no phenotypes (see Table 2 for a summary of tissue-specific deletion approaches in murine models).

Abrogation of KLF4 function has been well described following conditional deletion in epithelial tissues of the cornea [112,113,114,115,116], gastrointestinal tract [7,120,123,126,127,142,154], oral cavity [135], Sertoli cells [119] and skin [144]. In addition to epithelia, however, KLF4 also plays similar roles in driving differentiation of the hematopoietic system [121,131,132,133,136,137,161], endothelial cells [165], heart musculature [151,152,153], lymphatic system [143] and smooth muscle [141,162]. Overall, however, these minor phenotypic defects can generally be summarized in the loss of KLF4 leading to reduced, impaired or disorganized cellular differentiation and increased proliferation, self-renewal or multipotency, in keeping with the role of KLF4 as a stem-cell (Yamanaka) factor.

As impaired homeostasis is often a hallmark of the transition to malignancy, analyses of these disrupted physiological states may often provide clues to understanding KLF4 function. Of direct relevance to this review, however, is the fact that these processes typically also underpin the transition from a normal cell to malignancy, and therefore help to understand the processes and molecular pathways that may be de-regulated in both physiological and malignant states. A number of studies have indeed reported the presence of stronger phenotypes following conditional deletion, including a number of models that point to a critical, driving role in the progression to malignancy, such as increased incidence of spontaneously occurring squamous dysplasia [120,135], gastric tumors [130], and epithelial hyperplasia in the pancreas [138], lung [67], tongue, esophagus and forestomach [142]. These data would seem to indicate that KLF4 loss alone can certainly increase proliferation, but more importantly, as discussed in the next chapter, this loss typically serves to “sensitize” a cell or tissue towards far more substantial consequences when combined with a further insult, thereby comprising a classic “two-step” process towards disease states such as neoplasia.

## 9. KLF4 Loss and Secondary Insult—A Paradigm for the Induction of Malignancy?

Multiple studies have definitively shown that KLF4 loss substantially impacts on disease progression following inducement of a stressor. Typically, cancer incidence, age of onset or speed of progression is accelerated in well-established models of cancer, such as on the background of *Apc*/*Min*^+^ [129], Notch [132] or Ras [135,138] mutations or following DMBA/TPA induction [125]. However, loss of KLF4 also leads to increased axon regeneration of adult retinal ganglion cells following injury [155] and is neuroprotective in the experimental autoimmune encephalomyelitis (EAE) mouse model of multiple sclerosis [157]. These data dovetail nicely with the concept of KLF4 being an instigator of “genetic susceptibility”, that is, that predisposing genetic instability requires an environmental (or other genetic) trigger in order to effect change. Lessons from these other, non-cancer settings also correlate well with the “two-step” theory of cancer progression, in that multiple genetic mutations are typically required for neoplasia to arise from an otherwise healthy cell.

It is probable, however, that depending on which tissue *Klf4* is deleted in, together with the experimental paradigm being used, may lead to conflicting results about the nature of KLF4 function. For example, deletion of *Klf4* in perivascular smooth muscle cells (pvSMCs) within large arteries led to reductions in atheroma formation [145], however deletion of *Klf4* in endothelial cells significantly increased atherosclerosis progression in mice fed a high-fat diet [148]. As angiogenesis and maintenance of vascular tone are critical for malignancy, these findings suggest that KLF4 may impact not only on atherosclerosis, but more generally, on blood vessel homeostasis. Deletion of *Klf4* in smooth muscle surrounding arterioles led to reduced distal pulmonary arteriole muscularization, prevention of right ventricular hypertrophy and prevention of hypertension [162], yet endothelial cell deletion (following hypoxia) led to increased right ventricular and pulmonary artery pressures and increased right ventricular hypertrophy [149,150]. Such stressors indicate that even outside of cancer, environmental influences on KLF4 function substantially exacerbate KLF4 loss, and it is clear, therefore, that experimental conditions, choice of *Cre*-driver and the genetic backgrounds of mice may all act in concert with *Klf4* deletion to impact on disease progression.

These findings naturally also impact on the analysis of cellular process relevant for malignancy, in which the role of KLF4 using conditional mouse models has perhaps been best studied. Early experiments through conditional deletion in the intestine showed that KLF4 was integral for differentiation of goblet cells within the intestinal epithelium [108], with loss of KLF4 correlating with a concomitant increase in epithelial cell proliferation, defective maintenance of epithelial structure and integrity (as seen by mispositioning of Paneth cells in the walls of the villi of the small intestine [123,126], and increased migration in the small intestine, correlating with the cancerous process of metastasis. However, subsequent studies also showed that in the context of subsequent treatment of *Klf4*-deficient mice with a “stressor”, further phenotypes began to emerge. Typically using the *Villin-Cre* driver to effect intestinal deletion, studies showed that KLF4 loss was protective against development and progression of colitis-associated colorectal cancer (CAC) by guarding against genetic instability [7]. Additionally, these mice were also significantly less sensitive to Dextran Sodium Sulfate (DSS)-induced colitis, suggestive of a decreased sensitivity to tissue destruction as a consequence of inflammation [127]. These mice also presented with a significantly increased mortality following irradiation, likely due to an inability to adequately re-establish intestinal epithelial barriers through abrogated differentiation [128], again correlating with similar de-regulation of these processes in malignancy. Lastly, these mice also presented with significantly increased tumor formation following genetic mutation (*Apc*/*Min*^+^) or pharmacological treatment (azoxymethane) [129]. Together, these studies indicate that KLF4 is an important protective factor against hyperproliferation, de-regulation of tissue stability, and is essential for terminal differentiation.

However, *Klf4*-deletion has also been shown to either directly underpin cancer development or progression, or disrupt processes relevant for cancer development or progression, in non-epithelial tissues, typically (but not exclusively) in the presence of a genetic (or other) compounding stressor. Within the hematopoietic system, for example, KLF4 loss in malignancy leads to accelerated development of NOTCH1-induced T-cell Acute Lymphoblastic Leukemia (T-ALL) by promoting expansion of leukemia-initiating cells and impaired self-renewal and survival of chronic myeloid leukemia (CML) stem/progenitor cells, contributing to impaired maintenance of leukemia in a model of CML-like myeloproliferative neoplasia [131,132,133], and leading to significant reductions in metastasis to the lungs [136].

In non-malignant states, *Klf4*-loss leads to a decrease in numbers of pre-B cells in bone marrow, mature B cells in spleen [117], reduced NK cells in the blood and spleen [131], impaired differentiation and reduced proliferation of thymocytes [122] and enhanced neointima formation in response to vascular injury (caused by increased cellular proliferation) [118]. *Klf4* loss also contributes to the formation of both the vasculature and the lymphatic systems, where conditional deletion in endothelial cells (*VE-Cre* in the context of ApoE deletion acting as a compounding genetic stressor) promoted endothelial to mesenchymal transition (EndoMT) [150], whereas inducible deletion in lymphatic cells (using *Prox1-Cre* and Tamoxifen) led to defects in lymphatic branching morphogenesis and decreased lymphatic density [143], suggestive of abrogated morphology, differentiation and overall, angiogenesis.

Lastly, *Klf4* deletion within cardiac myocytes (using *Myh6-Cre* or *aMHC-Cre*) revealed the novel and surprising finding that KLF4 is essential for mitochondrial function. Conditional *Klf4* abrogation led to impaired mitochondrial biogenesis and maturation, reduced mitochondrial respiration and hyperacetylation of mitochondrial proteins [151,152,153]. As mitochondrial regulation of transcriptional circuits and signaling pathways is a critical—yet often underappreciated—component of cancer growth and survival [169], the finding that KLF4 normally regulates these crucial mitochondrial functions has opened up further avenues of research for better understanding this critical transcription factor.

## 10. Conclusions and Perspectives

In most cancers KLF4 appears to act as a tumor suppressor. However, the roles and regulation of KLF4 in cancer are complex and many aspects must be taken into consideration. The functioning of KLF4 is regulated at multiple levels: by regulation of transcription, alternative splicing, miRNA, post-translational modifications, subcellular localization, interactions with other molecules as well as by ubiquitination and subsequent proteasomal degradation [1]. In HCC the activity of KLF4 is regulated at the level of protein stability [51], alternative splicing [57], physical association with other proteins [56], as well as by various genetic and epigenetic mechanisms. In NSCLC the mechanisms of regulation of KLF4 activity include protein stability [71] and subcellular localization [72]. The latter is also important in prostate cancer [98]. Therefore simple experiments, for example measurements of transcript levels by microarray or Q-RT-PCR, or assays of protein levels by Western blotting, are insufficient to deliver valuable insights and may produce confusing results.

There are very important differences between tumor subtypes and stages. For example, in prostate cancer KLF4 is highly expressed in indolent tumors where it blocks malignant transformation and impedes tumor progression, while it is absent from aggressive prostate tumors [100]. This finding may prove to be very beneficial in the diagnosis of prostate cancer, as indolent tumors are usually harmless and do not require any treatment. Microenvironment is very important in the development of any type of cancer, and KLF4 plays a role in the microenvironment of prostate tumors [105].

Many of the original reports cited here state that KLF4 can be a therapeutic target in the treatment of cancer. However, it is very difficult to target transcription factors, especially those that lack ligand-binding domains and, unfortunately, KLF4 belongs to this category [1]. Nevertheless, KLF4 is important in regulating response to many known drugs [1]. These include cetuximab [23], cisplatin [19,43,72,84,102], gefitinib [79], glibenclamide [78], mesalazine [22] and Sijunzi decoction [20].

Many upstream regulators, downstream targets and associated signaling pathways have been implicated in the involvement of KLF4 in cancer. These include MAPK/JNK, TGF-β, GSK-3β, c-Jun, androgen receptor and other discussed here. It might be premature to speculate that KLF4 is a key regulator at the crossroads of signaling pathways relevant for cancer development. However, the evidence for significance of KLF4 in cancer is accumulating very rapidly, highlighting the critical function this transcription factor performs in the overall regulation of tissue homeostasis, cellular integrity, and progression towards malignancy.

In the future studies it will be essential to take into account all the concerns listed above. For example, it is insufficient to assay only the levels of KLF4 transcript, as the activity of KLF4 is also regulated by other mechanisms, which include alternative splicing and controlling the rate of KLF4 protein degradation. Thus Western blotting or other methods allowing for the quantification of KLF4 protein levels are necessary. Another very important aspect is the subcellular localization of KLF4, which is crucial for its function. Furthermore, it is vital to pay very close attention to cancer subtypes, which was made evident by the studies of prostate cancer. KLF4 is also very important in tumor microenvironment. Without addressing all of these issues it will not be possible to ascertain the importance of KLF4 in various cancer subtypes and, consequently, to establish whether KLF4 can serve as a cancer biomarker or a target for anti-cancer therapy in a particular cancer subtype.

## Figures and Tables

**Table 1:** Summary of the molecular mechanisms responsible for the involvement of KLF4 in the most common cancer types. Since KLF4 is a transcription factor, almost all of its downstream targets are regulated at the level of transcriptional initiation. Thus we only include statements “positive” and “negative” to indicate whether the transcription of this particular target gene is regulated positively or negatively by KLF4.

Cancer Type	Upstream Regulator/Downstream Target	Gene/Pathway	Molecular Mechanism	Reference
Colorectal cancer	upstream regulators	BMI1	methylation of KLF4 promoter	[25]
		miR-7-5p, miR-10b, miR-25-3p, miR-103/107, miR-130a, miR-135b, miR-153-1, miR-543	negative regulation by microRNA	[12,13,14,15,16,17,22]
	downstream targets	GINS4	negative	[5]
		NDRG2	positive	[4]
		tumor microenvironment	myeloid cell infiltration	[9]
Breast cancer	upstream regulators	AR, DYRK2	transcription of KLF4	[42]
		NFI-C	transcription of KLF4	[31]
		DDX3X	splicing of KLF4 primary transcript	[30]
		miR-7, miR-1233-3p	negative regulation by microRNA	[29,40,41]
		ATXN3	KLF4 protein degradation	[36]
		FBXO32	KLF4 protein degradation	[37]
	downstream targets	E-cadherin	positive	[32]
		LASS2	positive	[33]
		PFKP	positive	[39]
		S100A14	positive	[38]
Hepatocellular carcinoma	upstream regulators	EGFR	transcription of KLF4	[64]
		SET8	transcription of KLF4	[46]
		SF3B4	splicing of KLF4 primary transcript	[57]
		miR-9-5p, miR-10b, miR-18a, miR-124	negative regulation by microRNA	[52,53,54,55]
		TRAF7	KLF4 protein degradation	[51]
		DDX17	transcriptional transactivation activity of KLF4	[56]
	downstream targets	CD9, CD81	positive	[62]
		EGFR	positive	[64]
		EpCAM, CD133/Prominin-1	positive	[66]
		KLF11	negative	[55]
		MGLL	positive	[58,59]
		miR-31	positive	[61]
		P-cadherin	positive	[63]
		RYBP	positive	[60]
		SIRT4	positive	[46]
		Smad7	positive	[47]
Lung cancer	upstream regulators	SUR1, p70S6K, DNMT1	methylation of KLF4 promoter	[78]
		SIRT6, Snail	transcription of KLF4	[76]
		miR-25, miR-103, miR-145, miR-3120-5p	negative regulation by microRNA	[69,73,74,75]
		MALAT1, TRHDE-AS1	positive regulation by lncRNA	[74,75]
		USP10	KLF4 protein degradation	[71]
	downstream targets	β-catenin	negative	[79]
		β-catenin, c-Met	inhibition of binding between c-Met and β-catenin	[79]
		MMP2, PLAC8	negative	[70,77]
		TIMP3	positive	[71]
Gastric cancer	upstream regulators	CagA, TET1	methylation of KLF4 promoter	[92]
		LINC00673, EZH2, DNMT1	methylation of KLF4 promoter	[86]
		miR-32, miR-103, miR-135b-5p, miR-155	negative regulation by microRNA	[81,82,83,84,85]
		SNHG5	positive regulation by lncRNA	[82]
	downstream targets	iASPP	negative	[88]
		PODXL	negative	[89]
		STK33	negative	[87]
Prostate cancer	upstream regulators	KMT2D	methylation of KLF4 promoter	[103]
		LINC00673, DNMT1, DNMT3a, DNMT3b	methylation of KLF4 promoter	[96]
		AR	transcription of KLF4	[97]
		miR-7, miR-32-5p, miR-148-3p, miR-152-3p	negative regulation by microRNA	[94,95,102]
	downstream targets	AR	positive	[97]
		BIK	positive	[102]
		IGF2	positive	[104]
		miR-1	positive	[97]
		miR-7	positive	[94]
		tumor microenvironment	pro-inflammatory states	[105]

**Table 2:** Summary of animal models used to investigate KLF4 function.

Allele Name	Cells Targeted	Genetic Modification	Phenotype	Reference
Klf4tm1.1Khk		The floxed region encompassing exons 2 and 3 was excised from Klf4tm1Khk via cre-mediated recombination in the germline. FULL KO MOUSE	Defective goblet cell differentiation in colonic epithelium	[108]
Klf4tm1.1Khk	Neural Progenitor Cells (NPCs)	Nestin-Cre	Increased neurogenesis and reduced self-renewal in cortex.	[109]
Klf4tm1.1Khk	Fibroblasts	*Klf4*^−/−^ cells from lung	p21 mRNA expression reduced prior to birth. Ongoing cell proliferation after birth. Impaired myofibroblast differentiation at tips of alveoli.	[110]
Klf4tm1.1Khk		*Klf4*^+/−^/*Apc/Min*^+^	Increased incidence of intestinal adenomas.	
Klf4tm1Khk	Gastric mucosa (glandular)	Foxa3 YAC used to direct expression of Cre recombinase	Increased proliferation of gastric epithelia. Defective epithelial differentiation and mucin production No increased inflammation, intestinal metaplasia, dysplasia, or cancer.	[111]
Klf4tm1Khk	Corneal Epithelia	Pax6-Cre (Le-Cre) Krt12rtTA/rtTA/Tet-O-Cre	Corneal epithelial fragility and increased proliferation. Disrupted corneal epithelial cell identity Promotion of mesenchymal over epithelial cell identity. Defects in lens formation.	[112,113,114,115,116]
Klf4tm1Khk	B-cells	CD19-Cre	Decrease in numbers of pre-B cells in bone marrow and mature B cells in spleen.	[117]
Klf4tm1Khk	Tamoxifen inducible—model of vascular injury	ERT-Cre	Enhanced neointimal formation in response to vascular injury caused by increased cellular proliferation. Transient delay in repression of SMC differentiation markers in response to vascular injury.	[118]
Klf4tm1Khk	Sertoli cells	Anti Müllerian hormone (AMH)-Cre	Disorganized germinal epithelium and delayed lumen formation. Impaired apical secretion.	[119]
Klf4tm1Khk	Squamous epithelia of the tongue, esophagus, and forestomach	ED-L2 promoter of Epstein-Barr virus to drive Cre (ED-L2-Cre)	Increased basal cell proliferation and a delay in cellular maturation. Epithelial hypertrophy and subsequent dysplasia by 6 months of age.	[120]
Klf4tm1Khk	Myeloid cells	LysM-Cre	Critical in regulating M1/M2 macrophage polarization. Promotes M1 (pro-inflammatory) macrophage differentiation. Loss of klf4 in myeloid cells slows growth of subcutaneously transplanted prostate cancer cell line.	[121]
Klf4tm1Khk	CD4+ Th1 thymocytes (T-cells)	CD4-Cre	Modest reduction of thymocytes due to the reduced proliferation of double-negative (DN) thymocytes. Significant reduction of IL-17-expressing CD4+ T cells.	[105,122]
Klf4tm1Khk	Differentiated (adult) intestinal epithelial cells	KLF4/CreER (endogenous locus)	Increase in cell proliferation Increased number of goblet cells in small intestine. Mispositioning of Paneth cells along the small intestinal crypts	[123]
Klf4tm1Khk	Hair-follicle stem cells	KLF4/CreER (endogenous locus)	Bulge stem cell-enriched population decreased. Delayed cutaneous wound healing.	[124]
Klf4tm1Khk	Skin	KLF4/CreER (BAC)	Increased migration and adhesion of primary keratinocytes. Increased cell proliferation and skin carcinogenesis in DMBA/TPA skin cancer model	[125]
Klf4tm1Khk	Villus and crypt epithelial cells of the small and large intestine	Villin-Cre	Increased epithelial cell proliferation and migration in small intestine. Mispositioning of Paneth cells in SI Impaired goblet cell differentiation in colon Protective against development and progression of colitis-associated colorectal cancer (CAC) by guarding against genetic instability. Significantly less sensitive to Dextran Sodium Sulfate (DSS)-induced colitis. Significantly increased mortality following irradiation. Increased tumour formation following genetic mutation (ApcMin/+) or pharmacological treatment (azoxymethane)	[7,126,127,128,129]
Klf4tm1Khk	Antral mucosa cells (Stomach)	Villin-Cre	Increased gastric tumor development, exclusively in the lesser curvature of the antrum.	[130]
Klf4tm1Khk	Hemopoietic cells	Mx1-Cre Vav-iCre RosaCreER transduced with NOTCH1 retrovirus	Significant reduction of NK cells (NK1.1+ TCR-β−) in the blood and spleen. Increased apoptosis of CD27^+/−^ CD11b^+^ NK cells in the spleen. Accelerated development of NOTCH1-induced T-ALL by promoting expansion of leukemia-initiating cells. Impaired self-renewal and survival in CML stem/progenitor cells. Impaired maintenance of leukemia in a model of CML-like myeloproliferative neoplasia. De-repression of DYRK2.	[131,132,133]
Klf4tm1Khk	Osteoblasts/Osteoclasts	Col1α-Cre	Increased bone mass and enhanced bone formation. Significantly increased numbers of osteoclasts and osteoblasts.	[134]
Klf4tm1Khk	Oral cavity epithelia	K14-CreER	Dysplastic lesions, increased cell proliferation and abnormal differentiation in the tongue. Develop oral SCC following Ras activation	[135]
Klf4tm1Khk	Bone Marrow (esp. monocytes)	Rosa26-Cre ERFSP-1-Cre	Significantly reduced pulmonary metastasis. Compromised the generation of fibrocytes from MDSCs (myeloid-derived suppressor cells) Decreased expression of epithelial andTh2 cytokines. Impaired fibrocyte generation. Decreased airway hyperresponsiveness.	[136,137]
Klf4tm1Khk	Pancreas (esp. B-cells)	Pdx-Cre	Low incidence of hyperplasia in ductal epithelial cells. Reduced pancreatic intraepithelial neoplasia induced by mutant KrasG12D.	[138]
Klf4tm1Khk	Pancreatic cancer primary cell lines	AdCre viruses	Promoted acquisition of stem-like properties.	[139]
Klf4tm1Khk	Myeloid-derived CCR2+ suppressor cells	Fsp-1-Cre	Increased number of infiltrated lymphocytes in skin granule tissue. Significant hair and weight loss.	[140]
Klf4tm1Khk	Smooth Muscle Cells (inducible; adult)	SM22α-CreKI-YFP knockout (activated late in development)	Significant loss of multipotent adventitial Sca1+ cells. Premature death (by 4 weeks of age).	[141]
Klf4tm1Khk	Squamous epithelia of the tongue, esophagus, and forestomach	ED-L2-Cre	Hyperplastic esophageal epithelia with evidence of abnormal differentiation and stratification.	[142]
Klf4tm1Khk	Lung	Ad5CMVCre-eGFP (together with K-Ras activation)	Significantly increased lung tumorigenesis. Altered differentiation of lung tissue. Increased inflammation in lung.	[67]
Klf4tm1Khk	Developing lymphatic vessels	Prox1-CreERT2	Defects in lymphatic branching morphogenesis. Decreased lymphatic density.	[143]
Klf4tm1Khk	Epithelial tissue	Krt5-rtTA tetO-Cre	Differentiation defects in palmoplantar and tongue epithelia. Defects in filiform papilla structure.	[144]
Klf4tm1.1Khk	Perivascular Smooth Muscle Cells (SMCs) within large arteries	Myh11-CreERT2 in WT or Apoe^−/−^ mice	Reduced numbers of SMC-derived MSC- and macrophage-like cells. Decreased formation of a pre-metastatic niche and reduced metastasis. Reduction in atheroma size with concomitant increased stability. Significant cardiac dilatation. Impaired smooth muscle coverage of arteries. Arterial dilatation.	[145,146,147]
Klf4tm1Khk	Endothelial cells (with some leakiness in macrophages and lymphocytes)	VE-cadherin–Cre (on either wild-type or Apoe^−/−^ backgrounds)	Promoted endothelial to mesenchymal transition (EndoMT) Significantly enhanced development of atherosclerosis after 20 weeks of high-fat diet. Significantly increased right ventricular and pulmonary artery pressures (after hypoxia). More severe pulmonary vascular muscularization and right ventricular hypertrophy (after hypoxia).	[148,149,150]
Klf4tm1Khk	Cardiac myocytes	Myh6-Cre αMHC-Cre	Impaired mitochondrial biogenesis and maturation. Reduced mitochondrial respiration. Hyperacetylation of mitochondrial proteins. Cardiac dysfunction with aging or in response to pressure overload. Postnatal premature mortality. Altered ion channel (esp. K+) expression following Transverse Aortic Constriction-induced stress.	[151,152,153]
Klf4tm1Khk	Gastric epithelia and antral stem cells	Rosa26-Cre Lgr5-Cre	Increased proliferating cells and decreased pit mucous cells. Induction of MUC2 (goblet cell marker) in antrum.	[154]
Klf4tm1Khk	Retinal Ganglion Cells (RGCs).	Thy1-Cre AAV–GFPCre (adenovirus)	Increased axon growth both in vitro and after optic nerve injury in vivo. No difference in survival, but increased neurite length. Increased axon regeneration of adult RGCs. Prevented visual loss and increased neuroprotection in the chronic experimental autoimmune encephalomyelitis (EAE) mouse model of multiple sclerosis	[155,156,157]
Klf4tm1Khk	primordial germ cells (PGC) at E9.5–10.5	TNAP-Cre	No evident phenotype with regard to testicular histology, sperm maturation and fertility.	[158]
Klf4tm1Khk	Smooth Muscle	SM22α-Cre	Cardiac output significantly decreased. Marked growth retardation	[159]
Klf4tm1Khk	kidney glomerular podocytes	Podocin-Cre	Substantially exacerbated adriamycin-induced proteinuria (minimal phenotype otherwise).	[160]
Klf4tm1Khk	Conventional dendritic cells (cDCs)	Itgax-Cre	Impaired Th2 cell responses during challenge or infection. Selective loss of IRF4-expressing cDCs subsets	[161]
Klf4tm1Khk	Smooth Muscle	SMA-CreERT2	Prevented Pulmonary Hypertension (PH) and right ventricle (RV) hypertrophy. Reduced both distal pulmonary arteriole muscularization and PH.	[162]
Klf4tm1Khk	Endothelium	Cdh5(PAC)-CreERT2; Ccm1fl/fl; (double Ccm/Klf4 conditional deletion)	Reduction in number, size, and extension of the Cerebral Cavernous Malformations (CCM) in cerebellum. 70% reduction in cavernomas. 75% reduced mortality in Ccm1-deficient pups	[163]
Klf4tm1Khk	Retinal progenitor cells	Chx10-Cre	Increased thickness of axon bundles in the nerve fiber layer. No significant difference in cell number among any retinal cell types. No significant difference in photoreceptor layer thickness.	[164]
Klf4tm1Khk	Vasculature (endothelial cells); Model of Cerebral cavernous malformations (CCMs)	iECre; Krit1fl/fl	Reduced lesion formation. Rescued lethality.	[165]
Klf4tm1Khk	progenitor cells of the peripheral retina	α-Cre	Not essential for generation or differentiation of RGCs during retinogenesis.	[166]
Klf4tm1Khk	Pre-adipocytes Brown adipose tissue Back muscles	Retroviral-Cre/Adenoviral-cre (in vitro) Myf5-Cre (in vivo)	Not required for induction of brown adipose tissue. Musculature of back unaffected.	[167]
Klf4tm1Khk	Endocardium	Nfatc1-Cre	Required for remodeling of cardiac cushions to mature heart valves.	[168]
